# Strategies of strengthening mechanical properties in the osteoinductive calcium phosphate bioceramics

**DOI:** 10.1093/rb/rbad013

**Published:** 2023-02-17

**Authors:** Qipeng Li, Cong Feng, Quanle Cao, Wei Wang, Zihan Ma, Yonghao Wu, Tinghan He, Yangtian Jing, Wenxuan Tan, Tongxiao Liao, Jie Xing, Xiangfeng Li, Ye Wang, Yumei Xiao, Xiangdong Zhu, Xingdong Zhang

**Affiliations:** College of Chemical Engineering, Sichuan University, Chengdu 610065, China; National Engineering Research Center for Biomaterials, College of Biomedical Engineering, Sichuan University, Chengdu 610065, China; National Engineering Research Center for Biomaterials, College of Biomedical Engineering, Sichuan University, Chengdu 610065, China; College of Materials Science and Engineering, Sichuan University, Chengdu 610064, China; College of Materials Science and Engineering, Sichuan University, Chengdu 610064, China; National Engineering Research Center for Biomaterials, College of Biomedical Engineering, Sichuan University, Chengdu 610065, China; National Engineering Research Center for Biomaterials, College of Biomedical Engineering, Sichuan University, Chengdu 610065, China; College of Materials Science and Engineering, Sichuan University, Chengdu 610064, China; College of Materials Science and Engineering, Sichuan University, Chengdu 610064, China; College of Materials Science and Engineering, Sichuan University, Chengdu 610064, China; College of Materials Science and Engineering, Sichuan University, Chengdu 610064, China; National Engineering Research Center for Biomaterials, College of Biomedical Engineering, Sichuan University, Chengdu 610065, China; College of Chemical Engineering, Sichuan University, Chengdu 610065, China; National Engineering Research Center for Biomaterials, College of Biomedical Engineering, Sichuan University, Chengdu 610065, China; National Engineering Research Center for Biomaterials, College of Biomedical Engineering, Sichuan University, Chengdu 610065, China; National Engineering Research Center for Biomaterials, College of Biomedical Engineering, Sichuan University, Chengdu 610065, China

**Keywords:** CaP ceramics, strategy, mechanical property, osteoinductivity, enhancement

## Abstract

Calcium phosphate (CaP) bioceramics are widely applied in the bone repairing field attributing to their excellent biological properties, especially osteoinductivity. However, their applications in load-bearing or segmental bone defects are severely restricted by the poor mechanical properties. It is generally considered that it is challenging to improve mechanical and biological properties of CaP bioceramics simultaneously. Up to now, various strategies have been developed to enhance mechanical strengths of CaP ceramics, the achievements in recent researches need to be urgently summarized. In this review, the effective and current means of enhancing mechanical properties of CaP ceramics were comprehensively summarized from the perspectives of fine-grain strengthening, second phase strengthening, and sintering process optimization. What’s more, the further improvement of mechanical properties for CaP ceramics was prospectively proposed including heat treatment and biomimetic. Therefore, this review put forward the direction about how to compatibly improve mechanical properties of CaP ceramics, which can provide data and ideas for expanding the range of their clinical applications.

## Graphical Abstract

**Figure:**
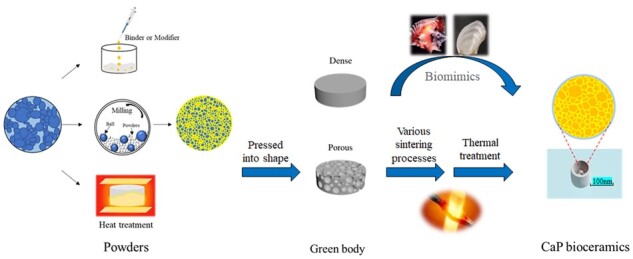

## Introduction

Bioceramics generally refer to ceramic materials with specific biological or physiological properties, which can be used to repair the diseased or impaired parts of musculoskeletal system. Generally, bioceramics are required to possess the following characteristics: good physicochemical stability, proper mechanical strength, biocompatibility and excellent affinity with biological tissues [1]. Bioceramics can be divided into bioinert ceramics and bioactive ceramics according to the strength of interfacial connection and fusion between bioceramics and bones [2, 3]. Nowadays, it is possible to design biomaterials with similar structural properties to natural bone by the means of bionics, which can avoid the risks and infections associated with autogenous and allogeneic bone grafts [4, 5]. How to endow biomaterials with biological functions to mobilize the self-healing functions of the human body and realize the regeneration of tissue defects has become the direction and frontier of regenerative medicine [6, 7]. Tissue inducing biomaterial, a kind of biomaterial through optimized designed material without adding any living cells and/or growth factors, could regenerate damaged tissues or organs, as described in the book ‘Definitions of Biomaterials for the Twenty-First Century’. As the pioneer, the discovery of osteoinductivity in calcium phosphate (CaP) bioceramics is fundamental and instructive, highlighting the potential to initiate a new generation of biomaterials [8].

Due to the similar mineral composition of human bone. They are generally considered as excellent bone grafts due to the good biocompatibility, bioactivity, osteoconductivity and osteoinductivity [9, 10]. CaP-based ceramics have been studied to replace human teeth and repair bone defects in the 1990s [11, 12]. CaP bioceramics mainly include hydroxyapatite (HA); tricalcium phosphate (TCP); and their combination biphase calcium phosphate (BCP); calcium-deficient HA (CDHA) etc. [13, 14]. Although CaP bioceramics have good bioactivity, they are still brittle materials with low fracture toughness, and have low impact resistance and relatively low tensile strength [15, 16]. Also, it is generally recognized that improvement of bioactivity properties of CaP ceramics is in contradiction with improvement of their mechanical performance [9, 17]. Generally, the high sintering temperature could increase the mechanical strength of CaP ceramics, but the relatively complete structure sintered at high temperature will debase the improvement of their bioactivity. How to find a balance between strengthening mechanical properties and enhancing biological properties of CaP ceramics is an important research direction of tissue engineering. Many researches have been done to promote the biological and mechanical characteristics of CaP bioceramics to make breakthroughs in load-bearing and segment bone defects [17–19]. In future, bioceramics will focus on optimized design of material itself, and adjustable micro-nano structure can improve its mechanical and biological properties, to further expand its clinical application scope [20, 21]. Up to now, there are few summaries and no similar review about enhancing mechanical properties of CaP bioceramics systematically and comprehensively. This work aims to summarize current and forward-looking research about enhancing mechanical properties of CaP ceramics, mainly including fine-grain strengthening, second phase strengthening, sintering process optimization and other treatments. In a word, it is essential for CaP bioceramics to further enhance mechanical strength while maintaining good biological performance.

## Strengthening of mechanical properties of CaP ceramics

**Figure 1.**
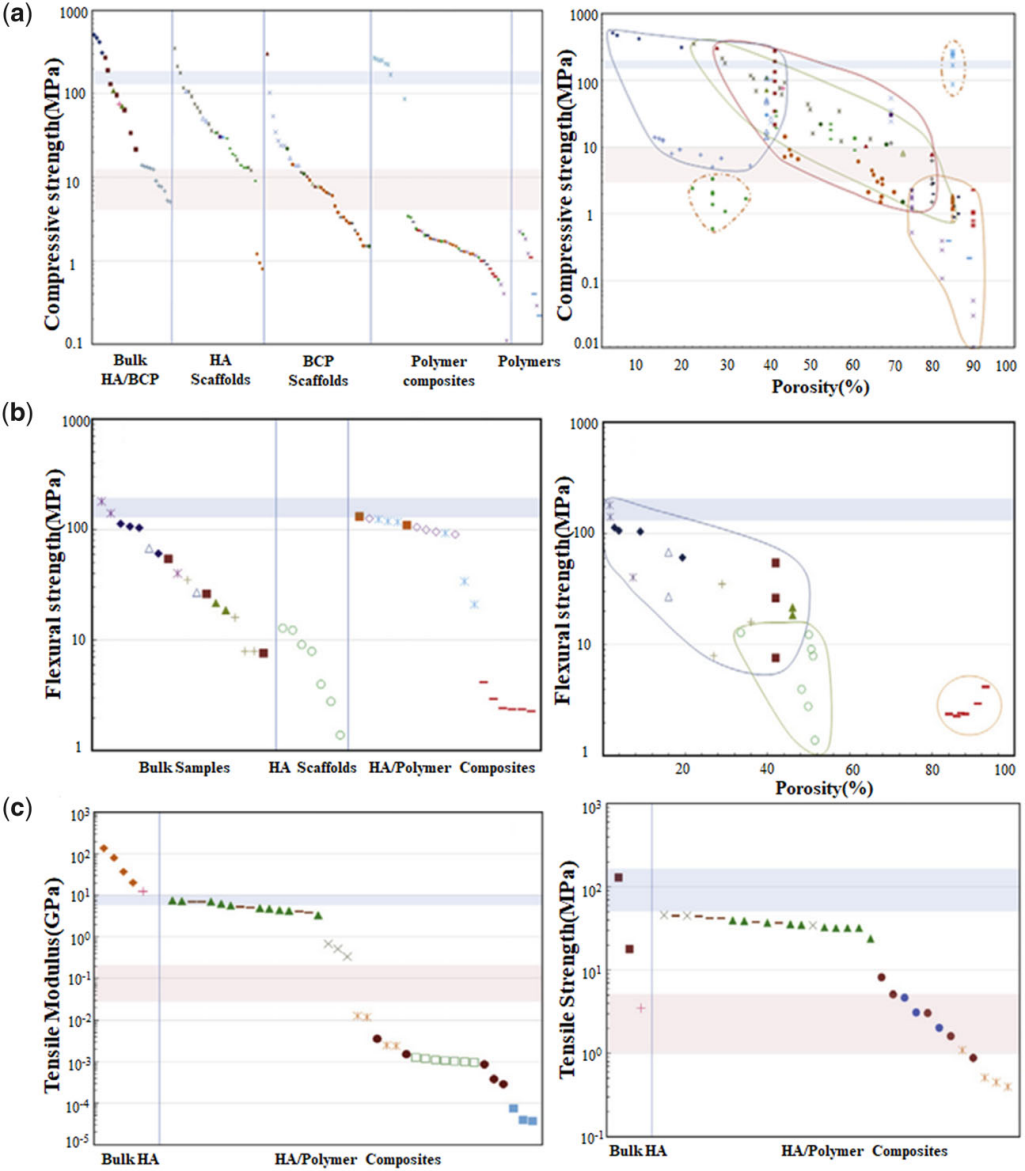
Mechanical properties of CaP ceramics (**a**, compressive strength; **b**, flexural strength; **c**, tensile modulus and strength) [16].

The main mechanical properties of CaP ceramics include compressive strength, flexural strength, tensile strength. As shown in Fig. 1 [16], mechanical properties of CaP ceramics are closely related to ceramics inherent porosity and doping phase. Bulk dense samples usually have better mechanical properties than scaffolds. For HA, flexural strength can be enhanced by preparing HA/polymer composites, but tensile strength and compressive strength will be reduced [16]. How to keep excellent mechanical properties while modifying CaP ceramics is difficult. Improving mechanical properties of CaP ceramics will offer great assistance in extending their application scopes while ensuring the biological properties.

### Strengthening by refining the grain size of CaP ceramics

The mechanical properties of CaP ceramics are closely related to their grain size and relative density. To strengthen mechanical properties of CaP ceramics, it is necessary to reduce grain size of CaP ceramics. Table 1 lists research results relating to the relationship between grain size and mechanical properties of CaP ceramics [17, 19, 22–28]. Different kinds of CaP ceramics could be prepared by refining the powder particles. The smaller the ceramic grain size was, the greater the hardness and yield strength of CaP ceramics would be. However, fracture toughness of conventional CaP ceramics was still not improved satisfactorily. This may be related to the fact that grain size has not reached the ultrafine nanometer level.

**Table 1.** Comparisons of the mechanical properties of CaP ceramics with different grain sizes

Composition	Average grain size (nm)	Vickers hardness (GPa)	Fracture toughness (MPa•m^1/2^)	Flexure strength (MPa)	Compressive strength (MPa)	Elastic modulus (GPa)	Ref.
HA	700	9.5	1.41	–	–	–	[22]
HA	200	11	–	–	–	145.4	[23]
HA/TCP	1000/3000	5	1.27	145.2	261.5	–	[19]
HA	1000	9.60	1.12	–	531.3	148.5	[24]
HA	130	4.19	1.3	–	–	–	[25]
HA	193	4.86	1.18	–	–	–	[26]
HA	190	7.80	1.92	–	–	–	[27]
BCP	375	4.90	1.11	–	–	–	[28]
BCP/CaO	250–637	1.23–2.20	1.89–4.17	25–35	36–93	1.25–3.25	[17]

Numerous studies have shown that CaP nanoceramics have high specific surface area and the enhanced mechanical properties compared with conventional CaP ceramics [29, 30]. The refinement of grains greatly increases the number of grain boundaries, which contributes to the slip between grain boundaries and makes the ceramics exhibit unique plasticity [31]. For nanoceramics, these advantages of similar structure to natural bone may result in higher biological activity and different biological characteristics [32, 33]. Some studies have reported that nanoceramics owned the remarkable ability to reduce apoptotic cell death, thus improving cell proliferation and cell activity associated with bone growth [34]. Therefore, preparation of CaP nanoceramics with excellent comprehensive properties is an important direction of bone tissue engineering. However, there are few mature technologies for preparing CaP nanoceramics. The main challenges lie in the synthesis of initial nano-sized powder and abnormal grain growth during high temperature sintering. The good initial properties of CaP powder are hard to control. In addition, there are some problems including the difficulty in controlling final geometry and uniform grain size of ceramics after sintering [35]. For example, the improper sintering process or sintering parameters could result in the uncontrolled growth of CaP grains. It is necessary to summarize certain rules by integrating relevant reports and optimizing the powder processing technology to reduce grain size of ceramics.

#### Strengthening CaP ceramics by refining the precursor

The ceramic preparation mainly consists of three continuous steps: synthesis of ceramic powders, molding and sintering of green body. To date, many methods have been used to prepare starting powders, including sol–gel processing [36], wet chemical precipitation [37], hydrothermal process [38], template method [39] and synthesis from biological sources [40]. By these methods, Ca-P nanoparticles with different structures and morphologies could be synthesized [29]. Among these methods, wet precipitation method and hydrothermal method were widely used, which can produce excellent precursors at nanometer size. Although nanocrystals synthesized by hydrothermal method had high crystallinity, small size and good shape, the yield was relatively low. Wet precipitation was a common method with simple operation, low operating temperature and high yield. Nonetheless, the size and the morphology of powder were easy to occur agglomeration [9]. And there were differences in morphology affecting the subsequent cell biological behaviors [41]. Optimizing process routes, reducing production costs and optimizing the synthesis of ceramics powder are potential and effective methods to facilitate improvement of biological properties and mechanical properties of CaP ceramics. There are few reports that the ultrafine CaP ceramics with grain size under 100 nm can be prepared. It was necessary to prepare dispersible nanoparticles with narrow distribution and high purity for the construction of nano-ceramics [42]. The relationship between the particle size of powder and grain size of the corresponding ceramics is summarized in Table 2 [35, 43–54]. The optimizing ultrafine CaP initial powder with high surface energy offered a great driving force for sintering process. Due to the small particle size, the atomic diffusion distance became short and diffusion coefficient became high, which benefits the preparation of nanoceramics. And the sintering temperature was much lower than the coarse particle materials [47, 55].

**Table 2.** Relationships between the initial powder size and the grain size of the representative ceramics

Powder composition	Application	Particle size of powder	Average grain size	Ref.
Al_2_O_3_	Bioinert ceramics	4.7/11.6/16.1 nm	34/70/164 nm	[43]
4YSZ	Biological coating	7.5 nm	30.3 nm	[47]
4YSZ		20–75nm	35–75 nm	[48]
(Hf_1/3_Ti_1/3_Zr_1/3_) B_2_	High temperature components	760 nm	6.62 μm	[49]
(Hf_1/3_Ta_1/3_Zr_1/3_) B_2_				
		350 nm	1.65 μm	
(Hf_1/3_Ta_1/3_Ti_1/3_) B_2_		260 nm	2.45 μm	
B_4_C(Ti-Al)	Wear-resistant parts	700/40 nm	700–800/200–300 nm	[50]
CoFe_2_O_4_	Magnetoelectric ceramic	350/50 nm	610–1920/460–833 nm	[51]
ZnO	Voltage-sensitive ceramics	1 μm/20 nm	17.33/3.63 μm	[52]
Gd_2_Zr_2_O_7_	Electronic structural elements	12.7/6.2 nm	15.8/7.2 nm	[53]
HA	Bioactive ceramics	50–100 nm	0.05–3μm	[35]
HA		200 nm	0.78–2.32 μm	[54]
β-TCP		0.5–3 μm/100 nm	5 μm/300 nm	[44]
β-TCP		80 nm	3 μm	[45]
BCP		30–80 nm	1–2 μm	[46]

4YSZ:4 mol% yttria stabilized zirconia.

The typical method for preparing nano-sized CaP powder is mainly divided into two categories. One is the optimization of *in situ* synthesis process for the ultrafine or nano-size powders. Lin [56] successfully synthesized monodisperse HA nanorods with narrow diameter distribution by hydrothermal microemulsion method. The bending strength and fracture toughness of the obtained HA bioceramics were obviously higher than those prepared by the normal powder. In a similar study [57], the average grain size of HA powder prepared by co-precipitation method was 50–70 nm. The final average grain size was 2–7 µm. The other preparing method of ultrafine powder is starting with synthesized initial powder and refining particle size or modifying powder through secondary treatment. Ball milling is the most common and effective method. Through ball milling, powder aggregates could be eliminated, and particle size of powder could be reduced. Ball milling can not only reduce the particle size, but also generate microstrains in the structure, which was beneficial to sintering of the ceramics [58, 59]. Studies have shown that nano HA powder could be obtained after vibrating ball milling. The maximum bending strength of dense HA ceramics was 15 MPa [60]. Like CaP ceramics, equiaxed α-Al_2_O_3_ nanoparticles with narrow particle size distribution and fine dispersion were obtained by directly ball-milling, solidification separation and gradient centrifugation [42]. HA ceramic powders also could be *in situ* synthesized by wet ball milling in the hydrothermal process, which could reduce agglomeration size of dry powder and maintain size of particles <22 nm [61]. However, contamination of grinding medium on the materials during ball milling was a serious problem [62]. Perhaps, we can explore an effective method combining ball milling, ultrasonic crushing and centrifugal separation to obtain the ultrafine powder with small particle size, narrow distribution and homogeneous dispersion.

#### Strengthening by powder modification treatment

In addition to refining the particle size of powder, we can also modify the initial powder via pretreatment. Common treatment processes mainly include powder sieving, activation and crystallization etc. Many studies show that it was necessary to pretreat ceramic powder by thermal treatment before sintering [24, 63, 64]. In addition, binder or polymerization agent was usually added to initial CaP powder to ensure that the dispersibility and strength of powder could be improved before sintering [65, 66]. What’s more, sintering properties of powder could be improved by adding some other active powder such as zirconia–alumina [67].

**Figure 2.**
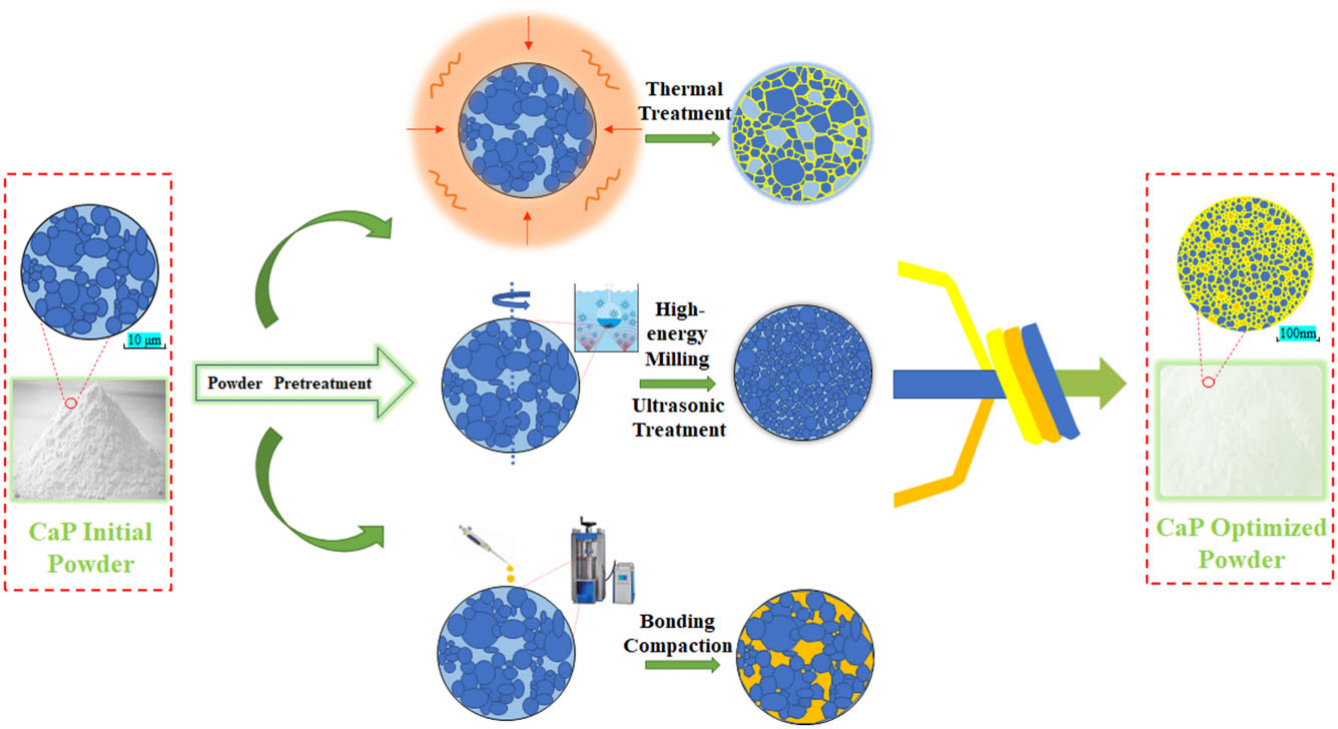
Schematic diagram of the treatment process of CaP ceramic powders.

Therefore, the study of CaP ceramics not only needs to investigate sintering and molding processes, the improvement of preceding powder was also beneficial [68]. As shown in Fig. 2, the initial crystal phase could be activated by heat treatment, which was conducive to sintering. Refining the initial particle size of powder by physical and chemical methods was beneficial to preparation of nanoceramics. And activator such as binder can make the powder have certain dispersity or caking property, which reduced agglomeration of powder. However, the research relating to such optimization and the mechanism has not been widely discussed. In the future, the pretreatment process of powder should be studied extensively.

### Optimizing sintering process to strengthen mechanical properties of CaP ceramics

Sintering process plays a vital role in the preparation of CaP ceramics because it needs to eliminate the pores existing inside billet and increase mechanical strength of the final products. The sintering process generally consisted of three successive stages: (i) in the initial stage, the formation and growth of interparticle neck could occur with light or without densification and continue until the relative density of fully dense material was ∼65%; (ii) in the intermediate stage, densification occurred by the shrinking of the pores. This stage covered a major part of sintering, and ended when the pores pinched off to become isolated, which corresponded to an increase in relative density to ∼90%; (iii) in the final stage of sintering, the isolated pores may disappear altogether, leaving a fully or nearly fully dense ceramic [69]. For CaP ceramics, grain growth occurs through grain boundary migration and many pores act as nails in grain boundaries. Hence, grain growth in the second stage was not obvious, and grain growth chiefly occurred in the last stage of sintering [19].

**Figure 3.**
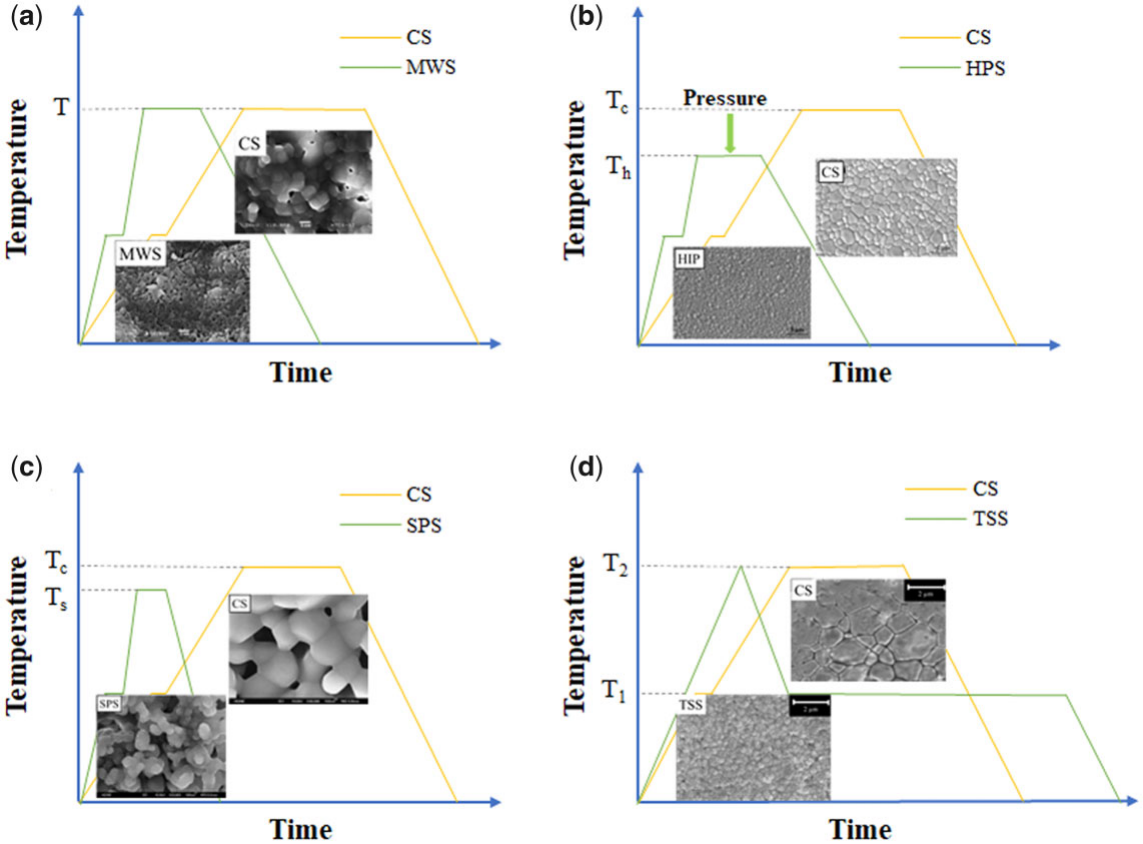
Schematic diagram of different sintering methods in CaP bioceramics (**a**, microwave sintering; **b**, pressure sintering; **c**, spark plasma sintering; **d**, TSS) [27, 64, 73, 74].

High temperature sintering was a high energy consumption and high-cost process [70]. How to control the abnormal grain growth during sintering was the key to improve toughness, mechanical strength and density of ceramics [71, 72]. As shown in Fig. 3 [27, 64, 73, 74], sintering processes employed in CaP ceramics mainly include conventional sintering, pressure sintering, spark plasma sintering (SPS), two-step sintering (TSS) etc. Different sintering methods own different sintering temperature curves and different equipment requirements. Selecting appropriate sintering process can save time and cost, enhancing mechanical properties of CaP ceramics. In sintering process of CaP ceramics, microwave sintering and TSS are more extensive and mature because of the universality for porous ceramics.

#### Microwave sintering

Microwave sintering is an economical and efficient sintering process used in fine ceramics such as Al_2_O_3_, Si_3_N_4_, PZT and some superconducting ceramics. The principle is using the dielectric loss of ceramic materials in microwave electromagnetic field to heat the whole material to sintering temperature and achieve denseness. Previous microwave sintering mainly focused on the preparation of porous or dense HA ceramics with nanocrystalline [75], then extended to β-TCP or BCP ceramics [25, 45]. Compared with conventional sintering, microwave sintering of porous HA/β-TCP ceramics had a significantly reduced grain size and more uniform structure, which may be caused by lower sintering temperature and shorter sintering time. Another was the uniform microstructure, which reduced the defects as the origin of cracks. The microwave sintered CaP ceramics had fine grains and high mechanical strength, which had a great prospect in the design of new bone substitutes [76].

Although microwave sintering could effectively optimize CaP ceramics microstructures, it still had some defects and is sometimes ineffective. On the one hand, it required special devices and was difficult to synthesis on large-scale. On the other hand, dielectric loss materials were often added in process of microwave heating to enhance heating efficiency, which may pollute CaP ceramics [9, 73].

#### Two-step sintering

TSS is also a promising method for obtaining CaP ceramics with high density and fine grains [77] without complex thermal programs. One was proposed by Chu [78], which needed thermal pretreatment sintering under low temperature, then the second phase sintering under high temperature. The other latest TSS method was proposed by Chen and Wang [79], which suppressed grain growth by heating the sample to a high temperature to achieve medium density firstly. Then it was cooled it and kept at a lower temperature until it was completely dense. Moreover, it should be paid attention to choose the appropriate *T*_1_ and *T*_2_ and their respective holding time for the different types. Although TSS was firstly applied to metal oxide ceramics, it was also suitable for CaP ceramics. TSS mainly inhibited the acceleration of grain growth of HA nanoparticles at the later stage of sintering [77]. The average grain size of the nearly fully dense HA ceramics prepared by conventional sintering was ∼1.7 μm [27]. However, with new TSS method, the ultimate grain size was ∼190 nm, and fracture toughness of specimen was increased by 95%. The two-step sintered BCP ceramics also showed that the microstructure was dense and uniform, with an average grain size of 375 nm [28]. The mechanical properties of two-step sintered BCP ceramics were better than those of conventional sintering.

TSS indicates that grain boundary migration and diffusion can be controlled by adjusting sintering curve, thus regulating ceramic microstructure. CaP ceramics with fine grain size and high density can be prepared by TSS method, but this method takes a long time and has low efficiency. What’s more, TSS has strict requirements on temperature, and optimum temperature parameters of different kinds of CaP ceramics are different.

The three kinds of sintering methods above have a wide range of applications and extensive research. Different sintering methods have different advantages and disadvantages and have different effects on mechanical properties. We have made a summary and induction for them, as shown in Table 3 [9, 20, 24–27, 45, 80, 81].

**Table 3.** Mechanical properties of CaP ceramics fabricated by various sintering processes

Sintering process	Temperature and time	Vickers hardness (GPa)	Fracture toughness (MPa•m^1/2^)	Elastic modulus (GPa)	Compressive strength (MPa)	Advantages and disadvantages [9]	Ref.
CS	1100°C, 1 h	4.55	–	–	917	*ADV*: Inexpensive devices, high yield, wide range of applications; *DISAD:* Unsuitable for nanoceramic fabrication	[80]
	1100°C, 3 h	6.3	0.88	–	–		[81]
	1150°C, 3 h	4.33	0.75	–	–		[26]
MWS	1100°C, 15 min	4.12	–	–	–	*ADV*: Rapid process, low energy cost, suitable for nanoceramic fabrication; *DISAD:* Expensive devices, low yield, difficult for large size of nanoceramic fabrication	[45]
	1230°C, 5 min	9.6	1.12	148. 5	531.3		[24]
	900–1200, 15 min	3.45–4.85	0.56–1.3	–	–		[25]
TSS	*T*_1_ = 1050°C, 0 h; *T*_2_ = 950°C, 20 h	4.86	1.18	–	–	*ADV*: Suitable for nanoceramic fabrication, high density, process stabilization; *DISAD:* Time-consuming, low efficiency, strict requirements for temperature accuracy	[26]
	*T*_1_ = 900°C, 0 h; *T*_2_ = 800, 20 h	7.8	1.92	–	–		[27]
	*T*_1_ = 1050°C, 0 h *T*_2_ = 950°C, 4 h	9.38	1.57	128.14	–		[20]

CS, conventional sintering; MWS, microwave sintering; TSS, two-step sintering.

#### Other optimized sintering processes

In addition to above sintering processes, pulsed current sintering [82], SPS [83] and hot pressing sintering [35] were also employed in sintering of CaP ceramics. Pulse current sintering can control fine structure because of its rapid heating and short sintering time, but it has low sintering efficiency and high energy consumption. Its sintering mechanism is controversial. SPS technology belongs to pulse electric current sintering, it also can achieve rapid sintering of nanoceramics. SPS can inhibit the growth of grain size, increase the density of substrate and promote growth of grain in the neck. Moreover, SPS could improve compressive strength and elastic modulus, providing a new way for the preparation of macroporous ceramics. However, the theory of sintering method is not completely clear, and this sintering technology cannot prepare large-size materials. Hot pressing sintering can prepare ceramics with high density, which can reduce the sintering temperature and shorten the sintering time. But this method is inefficient and costly, as well as has high requirements for equipment. It is only suitable for the preparation of dense ceramics, not suitable for porous ceramics. Therefore, the selection of appropriate sintering methods according to composition and porosity of the materials needs more serious attention when preparing CaP ceramics with high performances.

### Strengthening the CaP ceramics by whisker and second phase

#### Strengthening by whisker

**Figure 4.**
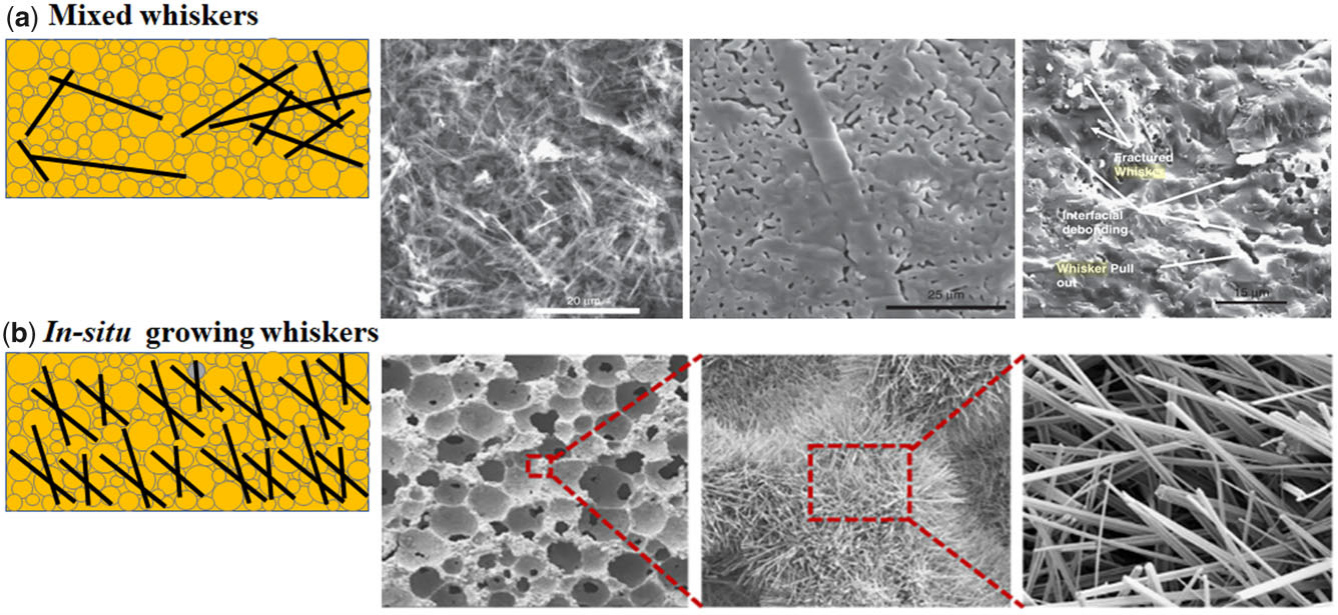
Sketch map illustrating the mixed whiskers (**a**) [30] and *in situ* growing whiskers (**b**) in CaP ceramics [87].

The brittleness is one of the fatal weaknesses of ceramics because crack is easy to spread and diffuse, which leads to final fracture of ceramics. In order to reduce cracks during the preparation of ceramics, addition of the second phase was able to regulate microstructure of ceramics to improve their mechanical properties [30]. Doping whisker is also widely used in improving mechanical strengths of CaP ceramics. The inorganic whisker material is an ideal toughening and reinforcing microfiber crystal, which can be added to the ceramics as a reinforcing phase. Whisker toughening is achieved by whisker in the ceramic to prevent crack propagation in the matrix, which has effects on whisker bridging, pulling out, crack deflection and micro-cracking etc. [84, 85]. Whiskers often existed in the form of rods in CaP ceramics, dispersing in the ceramic matrix, as shown in Fig. 4a [30]. Up to now, there are many studies on mechanical properties of CaP ceramic with whiskers reinforcement. Adding whiskers can restrain grain growth, improve sintering kinetics and retain the morphology of whisker in the final sintered ceramics. The mechanical properties of CaP ceramics were greatly improved by doping various whiskers [86]. However, the strength improvement of whisker reinforced ceramics was limited, which may be due to the random distribution and non-uniformity of whisker content in the ceramic matrix. Whisker cannot exist independently from the matrix, which greatly restricted enhancement of mechanical properties. In addition, the doping of whiskers was detrimental to biological properties, limiting its clinical application. Therefore, the latest study proposed *in situ* growth whiskers to solve the above problems [87]. *In situ* whisker growth of CaP ceramic was shown in Fig. 4b [87]. Because of the *in situ* growth of whiskers, the mechanical and biological properties of the obtained BCP ceramics could increase simultaneously. Therefore, *in situ* whiskers growth provides a prospective strategy for expanding application of CaP ceramics to satisfy the requirements of load-bearing bone repair.

#### Strengthening by the second phase doping

To be compatible with both mechanical and biological properties, doping the different second phase to strengthen the mechanical properties of CaP ceramics is also a popular method. The second phase of reinforcement can be doped with different performances. A lot of researches have been done about influence and mechanism of the second phase [88–90]. The study showed that compactness, sintering property and hardness of CaP ceramics were decreased with increase of boron content [91]. Metal elements such as zinc, magnesium and calcium were beneficial to human body and graphene with superior mechanical properties can maintain good biological activity while improving mechanical properties. Another result showed that the BCP doped with 4 mol%Zn had the better Vickers hardness and fracture toughness [92]. In addition, HA powders doped with Mg^2+^, Sr^2+^ and Zn^2+^ ions were synthesized and sintered [93], and the phase composition and microstructure of HA/β-TCP composites were affected by ion doping during sintering. Compared with the undoped HA ceramics, their flexural strength, hardness and Young’s modulus increased remarkably. Although the mechanical properties of CaP ceramics could be improved by the second phase, the introduction of the second phase would pollute ceramics more or less. Recently, the carbon material emerged such as graphene. In terms of the biocompatibility of carbonaceous structures, it is known to be well tolerated by the body without any foreign reaction. The fracture toughness of HA-rGO nanocomposites was 203% higher than that of pure HA [94]. Consequently, it was a very prospective strategy to incorporate graphene as a second phase reinforcement in HA, as it can boost mechanical performance of HA without weakening biological properties. In another review [95], the reinforcing effects of diverse carbonaceous structures in HA matrix were discussed. It was crucial to select a material with excepted biological properties, and then it was necessary to evaluate its overall strength and toughness. However, the doped second phase generally had only a single function, which made it more restricted in the field of biomaterials. Therefore, the multifunctional second phase would also be doped to prepare multiplex composites to further overcome the deficiency that single performance improvement of binary composites [96–98].

**Figure 5.**
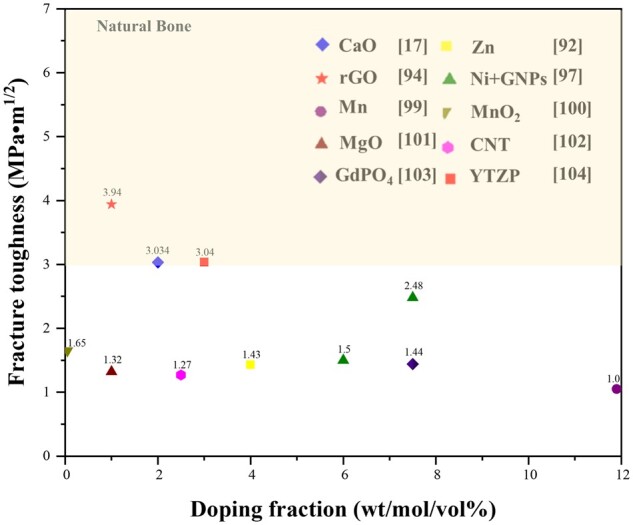
Diagram of the fracture toughness of CaP ceramics with second phase doping.

To sum up, the whisker reinforcement can improve mechanical properties of CaP ceramics to some degree, but the effect of improving ability is limited. In addition to whisker strengthening, we can choose the different second phases to raise expectations of different mechanical properties, by doping multiphase coupling effect ion and comprehensive to improve its mechanical and biological properties. However, some shortcomings still exist, such as its uniform distribution in matrix and the low porcelain density. At present, the friendly second phase such as graphene has great significance toughness, as shown in Fig. 5 [17, 92, 94, 97, 99–104]. Although there are few existing studies that have been able to break through fracture toughness of natural bone (3–7 MPa•m^1/2^), the above fine crystal strengthening, advanced sintering process and suitable doping process could increase the fracture toughness and mechanical strength of CaP ceramics, which can provide a way for the preparation of CaP ceramics with super mechanical properties for application in load-bearing bone defects.

### Other strategies

**Figure 6.**
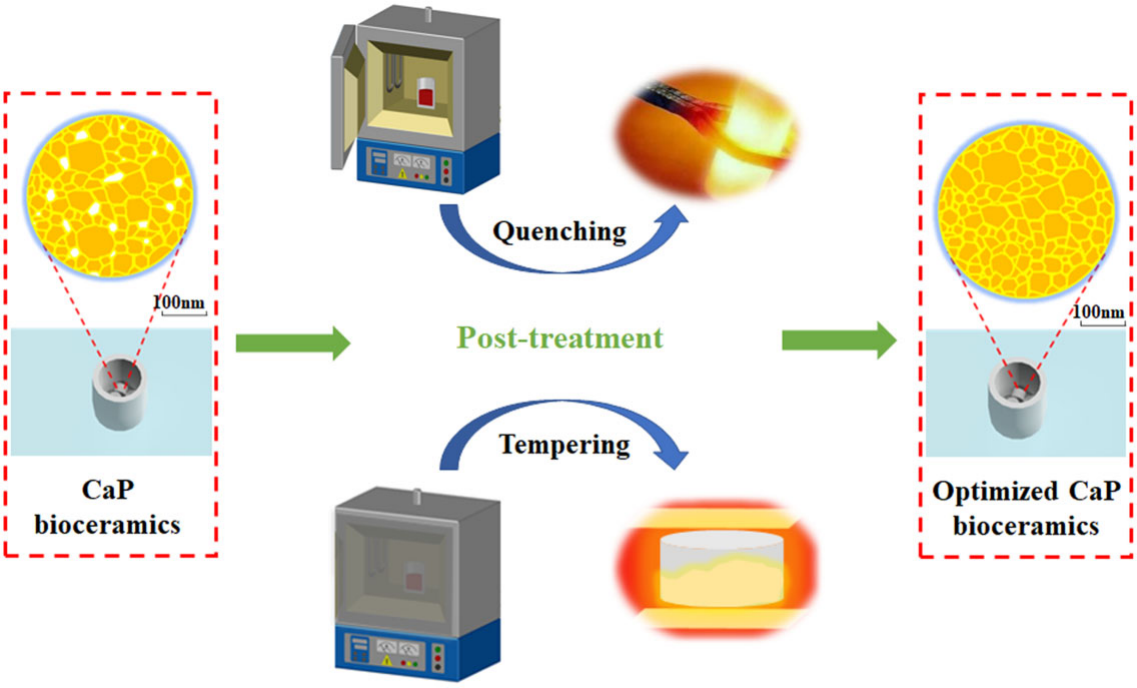
Schematic diagram of the prospective post-treatment process to strengthen the mechanical properties of CaP ceramics.

In addition to the above processes, there are some new advanced processes that could be used in ceramics in recent studies. Generally, heat treatment is a post-processing technology which can obtain expected structure and properties of the obtained materials. Heat treatment generally includes heating, heat preservation, cooling three processes. To improve mechanical properties of aluminum matrix composites, precipitation hardening process was usually used in heat treatment of aluminum matrix composites. After heat treatment, the hardness and tensile strength of the composites were increased [105]. Bismuth sodium titanate piezoelectric ceramics have been prepared by quenching method. The results showed that the depolarization temperature and mechanical strength could be improved by controlling quenching rate [106]. Quenching has also been used to improve properties of glass ceramics. The glass-ceramics obtained by optimized heat treatment had the highest density and dielectric constant [107]. After sintering, the sintered body should be taken out of the furnace immediately, because the quenching of oxide ceramics can reach a higher density than that in the furnace with furnace cooling, which was related to the cast effect of the outer layer of material under the rapid cooling. And the rapid cooling of ceramics produced compression effect of the outer layer of the material [59, 108]. Heat treatment process was also widely used to enhance or improve mechanical properties of materials even in biological applications [109–111]. For CaP ceramics, we can apply these advanced heat treatments after sintering. A recent study pointed out [112] that post-heat treatment of CaP ceramics would lead to grain growth and grain boundary migration, and the mechanical properties of the heat-treated ceramics were improved remarkably. By using these post-treatment methods, CaP bioceramics with high density and improved mechanical properties could be obtained potentially. However, the application of heat treatment in CaP bioceramics still remains to be further studied to break through the limitations of cracks and brittle fractures. Some studies have confirmed the occurrence of some problems during heat treatment. Crack generation and propagation phenomenon in the process of quenching were observed in alumina ceramics [113]. In addition, after thermal shock quenching, there would be a large amount of residual stress in ceramics [114], which was not beneficial to improvement of mechanical properties of CaP ceramics, and microcracks will also affect its biological properties. Although thermal shock has quite high requirements, it is expected to further improve CaP ceramics’ mechanical properties by adjusting process and reducing impact force after ceramics have a certain impact resistance. In future, through the theoretical analysis of related process, we can reduce quenching rate, using air quenching and low temperature quenching to suppress and avoid generation of cracks. The inner residual stress of ceramic could be eliminated by subsequent heat treatment at low temperature tempering. As shown in Fig. 6, the density and strength of sintered CaP ceramics can be improved by post-treatment.

Nowadays, more and more researches began to shift to the direction of biomimetic biomaterials. Natural materials exhibit the exceptional mechanical performance relying on their complex hierarchical structures at multiple length scales [115]. Through the biomimetic method, the hierarchical ‘brick-and-mortar’ structure in the bulk artificial nacre induced the enhanced both strength and toughness. The mechanical superiority of the fabricated bulk artificial nacre endowed it with great potential for future applications [116]. In the field of CaP ceramics, bionics is still applicable. CaP biomaterials fabricated by biomimetic routes possess distinct features compared to conventional Ca-P ceramics, such as a high specific surface area and nanometric crystal size [117]. By using biomimetic synthesis, a three-level hierarchical CaP/collagen/HA scaffold for bone tissue engineering was developed and exhibited a similar structure and composition to natural bone tissues. Furthermore, this three-level hierarchical biomimetic scaffold showed enhanced mechanical strength compared with pure porous CaP scaffolds [118]. In addition, it has been reported that the combination of cellulose can not only improve the strengths and modulus of the HA/cellulose composites, but also provide nucleation sites for calcium ion deposition through the phosphorylation modification [119]. Recently, a gelatin–CaP nanocomposite was synthesized by an efficient and cost-effective double-diffusion biomimetic approach [120]. The obtained scaffolds had the ability of bone regeneration while improving the compressive strength, which had been successfully applied in the regeneration of cranial bone defects. In summary, the preparation of CaP ceramics with excellent mechanical properties by biomimetic methods can better match the changes of the implanted environment, which will become an increasingly popular research direction.

This part is not limited to the strengthening strategies mentioned above. All methods that can improve the mechanical properties of CaP bioceramics could be considered. For example, in order to improve the mechanical properties such as compressive strength and compressive modulus and maintain the desirable bioactivity, the open micropores of the struts were infiltrated with poly (lactic-co-glycolic acid) to achieve an interpenetrating bioactive ceramic/biodegradable polymer composite structure [121]. Some other studies also constructed polydopamine nanolayer [122, 123] and polyethyleneimine/heparin nanogel [124] on the surfaces of Ca-P bioceramics to enhance their osteoinductivity and osteogenicity, but the mechanical enhancement is limited. With the development of science and technology, there are bound to be some advanced technologies and methods in the future, which can better expand the application of CaP bioceramics with enhanced mechanical properties in bone tissue engineering and regenerative medicine.

## Conclusion and prospect

Starting from ceramic powders, we can refine initial powders and take modification treatments, so that the phenomenon of agglomeration and non-uniform can be reduced. In terms of sintering process, mechanical properties of CaP ceramics could be enhanced by using microwave sintering. The abnormal growth of ceramic grain can be effectively restrained by the TSS. At the same time, the reinforced CaP ceramics by doping whiskers or the second phase can be prepared to meet different requirements. By referring to other methods of strengthening mechanical properties of ceramics, it is expected to develop some new methods and break through limitations of existing methods. Improving mechanical properties of CaP ceramics while maintaining biological activity has always been the goal of current research efforts. It is expected to break through defects of mechanical properties of traditional CaP ceramics via fine-grain strengthening, sintering process optimization and doping strengthening. Meanwhile, exploring new technology like heat treatment is also necessary. What’s more, it is potential to learn new processing technology from other kinds of ceramics and use bionic methods. All kinds of effective processes on improving mechanical properties of CaP ceramics can be referred in future. We can further undertake the interdisciplinarity and find a generality to enhance mechanical properties with little damage to biological activity of CaP ceramics, then being applied to load-bearing bone repairing field finally.

## References

[1] Hench LL. Bioceramics. J Am Ceram Soc 1998;81:1705–28.

[2] D’Antonio J , Capello W, Manley M, Bierbaum B. New experience with alumina-on-alumina ceramic bearings for total hip arthroplasty. J Arthroplasty 2002;17:390–7. 12066265 10.1054/arth.2002.32183

[3] Hench LL. Bioceramics: from concept to clinic. J Am Ceram Soc 1991;74:1487–510.

[4] Banwart JC , Asher MA, Hassanein RS. Iliac crest bone graft harvest donor site morbidity: a statistical evaluation. Spine 1995;20:1055–60. 7631235 10.1097/00007632-199505000-00012

[5] Khan SN , Cammisa FP, Sandhu HS, Diwan AD, Girardi FP, Lane LM. The biology of bone grafting. J Am Acad Orthop Surg 2005;13:77–86. 15712985

[6] Li X , Song T, Chen X, Wang M, Yang X, Xiao Y, Zhang X. Osteoinductivity of porous biphasic calcium phosphate ceramic spheres with nanocrystalline and their efficacy in guiding bone regeneration. ACS Appl Mater Inter 2019;11:3722–36. 10.1021/acsami.8b18525 30629405

[7] Li X , Wang Y, Chen F, Chen X, Xiao Y, Zhang X. Design of macropore structure and micro-nano topography to promote the early neovascularization and osteoinductivity of biphasic calcium phosphate bioceramics. Mater Des 2022;216:110581.

[8] Feng C , Wu Y, Li Q, He T, Cao Q, Li X, Xiao Y, Lin J, Zhu X, Zhang X. A novel Hollow-Tube-Biphasic-Whisker-Modified calcium phosphate ceramics with simultaneously enhanced mechanical strength and osteogenic activity. Adv Funct Mater 2022;32:2204974.

[9] Hong YL , Fan HS, Li B, Guo B, Liu M, Zhang X. Fabrication, biological effects, and medical applications of calcium phosphate nanoceramics. Mater Sci Eng R Rep 2010;70:225–42.

[10] Tang Z , Li X, Tan Y, Fan H, Zhang X. The material and biological characteristics of osteoinductive calcium phosphate ceramics. Regen Biomater 2018;5:43–59. 29423267 10.1093/rb/rbx024 PMC5798025

[11] Langer R , Vacanti JP. Tissue engineering. Science 1993;260:920–6. 8493529 10.1126/science.8493529

[12] Ripamonti U. The morphogenesis of bone in replicas of porous hydroxyapatite obtained from conversion of calcium carbonate exoskeletons of coral. J Bone Jt Surg Ser A 1991;73:692–706. 1675218

[13] Dorozhkin SV. Calcium orthophosphates. J Mater Sci 2007;42:1061–95.

[14] Vallet-Regí M , González-Calbet JM. Calcium phosphates as substitution of bone tissues. Prog Solid State Chem 2004;32:1–31.

[15] Groot KD. Bioceramics consisting of Cecil phosphate salts. Biomaterials 1980;1:47–50. 7470552 10.1016/0142-9612(80)90059-9

[16] Wagoner Johnson AJ , Herschler BA. A review of the mechanical behavior of CaP and CaP/polymer composites for applications in bone replacement and repair. Acta Biomater 2011;7:16–30. 20655397 10.1016/j.actbio.2010.07.012

[17] Wang Y , Wang M, Chen F, Feng C, Chen X, Li X, Xiao Y, Zhang X. Enhancing mechanical and biological properties of biphasic calcium phosphate ceramics by adding calcium oxide. J Am Ceram Soc 2021;104:548–63.

[18] Yousefi AM , Oudadesse H, Akbarzadeh R, Wers E, Lucas-Girot A. Physical and biological characteristics of nanohydroxyapatite and bioactive glasses used for bone tissue engineering. Nanotechnol Rev 2014;3:527–52.

[19] Hu X , Zhang W, Hou D. Synthesis, microstructure and mechanical properties of tricalcium phosphate-hydroxyapatite (TCP/HA) composite ceramic. Ceram Int 2020;46:9810–6.

[20] Wu Y , Cao Q, Niu M, Feng C, Li X, Zhu X, Zhang X. Comparative studies on micromechanical properties and biological performances in hydroxyapatite ceramics with micro/nanocrystalline. J Am Ceram Soc 2022;105:742–56.

[21] Zhang K , Fan Y, Dunne N, Li X. Effect of microporosity on scaffolds for bone tissue engineering. Regen Biomater 2018;5:115–24. 29644093 10.1093/rb/rby001 PMC5887944

[22] Tolouei R , Ramesh S, Tan CY, Amiritan M, Teng WD. Effect of grain size on vickers microhardness and fracture toughness in calcium phosphate bioceramics. Appl Mech Mater 2011;83:237–43.

[23] Ievlev VM , Kostyuchenko AV, Kochlar GS, Putlyaev VI. Structure and nanohardness of compact hydroxyapatite-based ceramics. Inorg Mater 2019;55:1054–60.

[24] Thuault A , Savary E, Hornez JC, Moreau G, Descamps M, Marinel S, Leriche A. Improvement of the hydroxyapatite mechanical properties by direct microwave sintering in single mode cavity. J Eur Ceram Soc 2014;34:1865–71.

[25] Veljović D , Zalite I, Palcevskis E, Smiciklas I, Petrović R, Janaćković D. Microwave sintering of fine grained HAP and HAP/TCP bioceramics. Ceram Int 2010;36:595–603.

[26] Lin K , Chen L, Chang J. Fabrication of dense hydroxyapatite nanobioceramics with enhanced mechanical properties via two-step sintering process. Int J Appl Ceram Technol 2012;9:479–85.

[27] Mazaheri M , Haghighatzadeh M, Zahedi AM, Sadrnezhaad SK. Effect of a novel sintering process on mechanical properties of hydroxyapatite ceramics. J Alloys Compounds 2009;471:180–4.

[28] Lukić M , Stojanović Z, Skapin SD, Macek-Krzmanc M, Mitrić M, Marković S, Uskoković D. Dense fine-grained biphasic calcium phosphate (BCP) bioceramics designed by two-step sintering. J Eur Ceram Soc 2011;31:19–27.

[29] Sadat-Shojai M , Khorasani MT, Dinpanah-Khoshdargi E, Jamshidi A. Synthesis methods for nanosized hydroxyapatite with diverse structures. Acta Biomater 2013;9:7591–621. 23583646 10.1016/j.actbio.2013.04.012

[30] Bose S , Banerjee A, Dasgupta S, Bandyopadhyay A. Synthesis, processing, mechanical, and biological property characterization of hydroxyapatite whisker-reinforced hydroxyapatite composites. J Am Ceram Soc 2009;92:323–30.

[31] Dorozhkin SV. Nanosized and nanocrystalline calcium orthophosphates. Acta Biomater 2010;6:715–34. 19861183 10.1016/j.actbio.2009.10.031

[32] Zhao R , Chen S, Yuan B, Chen X, Yang X, Song Y, Tang H, Yang X, Zhu X, Zhang X. Healing of osteoporotic bone defects by micro-/nano-structured calcium phosphate bioceramics. Nanoscale 2019;11:2721–32. 30672553 10.1039/c8nr09417a

[33] Sutthavas P , Habibovic P, Van Rijt SH. The shape-effect of calcium phosphate nanoparticle based films on their osteogenic properties. Biomater Sci 2021;9:1754–66. 33433541 10.1039/d0bm01494j

[34] Li B , Guo B, Fan H, Zhang X. Preparation of nano-hydroxyapatite particles with different morphology and their response to highly malignant melanoma cells in vitro. Appl Surf Sci 2008;255:357–60.

[35] Veljović D , Jokić B, Petrović R, Palcevskis E, Dindune A, Mihailescu IN, Lanaćković D. Processing of dense nanostructured HAP ceramics by sintering and hot pressing. Ceram Int 2009;35:1407–13.

[36] Algueró M , Ferrer P, Vila E, Iglesias JE, Castro A. Bi_4_Ti_3_O_12_ ceramics from powders prepared by alternative routes: wet no-coprecipitation chemistry and mechanochemical activation. J Am Ceram Soc 2006;89:3340–7.

[37] Tilkin RG , Mahy JG, Régibeau N, Grandfils C, Lambert SD. Optimization of synthesis parameters for the production of biphasic calcium phosphate ceramics via wet precipitation and Sol-Gel process. ChemistrySelect 2019;4:6634–41.

[38] Toyama T , Nakashima K, Yasue T. Hydrothermal synthesis of β-tricalcium phosphate from amorphous calcium phosphate. J Jpn Ceram Soc 2002;110:716–21.

[39] Bastakoti BP , Inuoe M, Yusa S, Liao SH, Wu KC, Nakashima K, Yamauchi Y. A block copolymer micelle template for synthesis of hollow calcium phosphate nanospheres with excellent biocompatibility. Chem Commun 2012;48:6532–4. 10.1039/c2cc32279j 22622697

[40] Deb P , Deoghare AB. Effect of acid, alkali and alkali-acid treatment on physicochemical and bioactive properties of hydroxyapatite derived from *Catla catla* fish scales. Arab J Sci Eng 2019;44:7479–90.

[41] Salma K , Berzina-Cimdina L, Borodajenko N. Calcium phosphate bioceramics prepared from wet chemically precipitated powders. Process Appl Ceram 2010;4:45–51.

[42] Li L , Pu S, Liu Y, Zhao L, Ma J, Li J. High-purity disperse α-Al_2_O_3_ nanoparticles synthesized by high-energy ball milling. Adv Powder Technol 2018;29:2194–203.

[43] Dong Y , Yang H, Zhang L, Li X, Ding D, Wang X, Li J, Li J, Chen IW. Ultra-uniform nanocrystalline materials via two-step sintering. Adv Funct Mater 2021;31:1–9.

[44] Lin K , Chang J, Lu J, Wu W, Zeng Y. Properties of β-Ca_3_(PO_4_)_2_ bioceramics prepared using nano-size powders. Ceram Int 2007;33:979–85.

[45] Mirhadi B. Microwave sintering of nano size powder β-TCP bioceramics. Sci Sinter 2014;46:185–93.

[46] Zhou C , Xie P, Chen Y, Fan Y, Tan Y, Zhang X. Synthesis, sintering and characterization of porous nano-structured CaP bioceramics prepared by a two-step sintering method. Ceram Int 2015;41:4696–705.

[47] Drazin JW , Wollmershauser JA, Ryou H, Wolak MA, Gorzkowski EP. Pressureless low temperature sintering of nanocrystalline zirconia ceramics via dry powder processing. J Am Ceram Soc 2020;103:60–9.

[48] Jiang K , Liu S, Wang X. Phase stability and thermal conductivity of nanostructured tetragonal yttria-stabilized zirconia thermal barrier coatings deposited by air-plasma spraying. Ceram Int 2017;43:12633–40.

[49] Zhang W , Zhang Y, Guo WM, Xu L, You Y, Sun SK, Lin HT, Wu SH. Powder synthesis, densification, microstructure and mechanical properties of Hf-based ternary boride ceramics. J Eur Ceram Soc 2021;41:3922–8.

[50] Ojalvo C , Zamora V, Moreno R, Guiberteau F, Ortiz AL. Transient liquid-phase assisted spark-plasma sintering and dry sliding wear of B_4_C ceramics fabricated from B_4_C nanopowders. J Eur Ceram Soc 2021;41:1869–77.

[51] Perdomo F , Zabotto CP, Garcia FL, Kiminami D. R. Effect of the CoFe_2_O_4_ initial particle size when sintered by microwave on the microstructural, dielectric, and magnetic properties. Int J Appl Ceram Technol 2019;16:2073–84.

[52] Liu W , Zhang L, Kong F, Wu K, Li S, Li J. Enhanced voltage gradient and energy absorption capability in ZnO varistor ceramics by using nano-sized ZnO powders. J Alloys Compounds 2020;828:154252.

[53] Huang Z , Deng J, Wang H, Zhang Y, Duan J, Tang Z, Cao Z, Qi J, He D, Lu T. Fast low-temperature densification of translucent bulk nanograin Gd_2_Zr_2_O_7_ ceramics with average grain size below 10 nm. J Alloys Compounds 2020;830:154617.

[54] Song J , Liu Y, Zhang Y, Jiao L. Mechanical properties of hydroxyapatite ceramics sintered from powders with different morphologies. Mater Sci Eng A 2011;528:5421–7.

[55] Cao L , Zhang C, Huang J. Synthesis of hydroxyapatite nanoparticles in ultrasonic precipitation. Ceram Int 2005;31:1041–4.

[56] Lin K , Chang J, Cheng R, Ruan M. Hydrothermal microemulsion synthesis of stoichiometric single crystal hydroxyapatite nanorods with mono-dispersion and narrow-size distribution. Mater Lett 2007;61:1683–7.

[57] Kong LB , Ma J, Boey F. Nanosized hydroxyapatite powders derived from coprecipitation process. J Mater Sci 2002;37:1131–4.

[58] Kostov-Kytin VV , Dyulgerova E, Ilieva R, Petkova V. Powder X-ray diffraction studies of hydroxyapatite and β-TCP mixtures processed by high energy dry milling. Ceram Int 2018;44:8664–71.

[59] Morozova LV. Mechanochemical activation of precursor powders for the preparation of dense Al_2_O_3_-ZrO_2_〈Y_2_O_3_〉 nanoceramics. Inorg Mater 2019;55:295–301.

[60] Raksujarit A , Pengpat K, Rujijanagul G, Tunkasiri T. Processing and properties of nanoporous hydroxyapatite ceramics. Mater Des 2010;31:1658–60.

[61] Chesley M , Kennard R, Roozbahani S, Kim SM, Kukk K, Mason M. One-step hydrothermal synthesis with *in situ* milling of biologically relevant hydroxyapatite. Mater Sci Eng C 2020;113:110962. 10.1016/j.msec.2020.110962 32487383

[62] Titorenkova R , Dyulgerova E, Petkova V, Ilieva R. Carbonation and dehydroxylation of apatite during high energy milling of biphasic Ca-phosphate ceramics. Ceram Int 2019;45:7025–33.

[63] Han JK , Song HY, Saito F, Lee BT. Synthesis of high purity nano-sized hydroxyapatite powder by microwave-hydrothermal method. Mater Chem Phys 2006;99:235–9.

[64] Zhang F , Lin K, Chang J, Lu J, Ning C. Spark plasma sintering of macroporous calcium phosphate scaffolds from nanocrystalline powders. J Eur Ceram Soc 2008;28:539–45.

[65] Safronova TV , Putlyaev VI. Powder systems for calcium phosphate ceramics. Inorg Mater 2017;53:17–26.

[66] Ahmed MA , Mansour SF, El-Dek SI, Abd-Elwahab SM, Ahmed MK. Characterization and annealing performance of calcium phosphate nanoparticles synthesized by co-precipitation method. Ceram Int 2014;40:12807–20.

[67] Kong YM , Bae CJ, Lee SH, Kim HW, Kim HE. Improvement in biocompatibility of ZrO_2_-Al_2_O_3_ nano-composite by addition of HA. Biomaterials 2005;26:509–17. 15276359 10.1016/j.biomaterials.2004.02.061

[68] Liu D , Wu Y, Wu H, Li X, Yang X, Zhu X, Zhang X. Effect of process parameters on the microstructure and property of hydroxyapatite precursor powders and resultant sintered bodies. Int J Appl Ceram Technol 2019;16:444–54.

[69] Champion E. Sintering of calcium phosphate bioceramics. Acta Biomater 2013;9:5855–75. 23212081 10.1016/j.actbio.2012.11.029

[70] Nakashima Y , Razavi-Khosroshahi H, Takai C, Fuji M. Non-firing ceramics: effect of adsorbed water on surface activation of silica powder via ball milling treatment. Adv Powder Technol 2019;30:1160–4.

[71] Ramesh S , Tan CY, Bhaduri SB, Teng WD, Sopyan I. Densification behaviour of nanocrystalline hydroxyapatite bioceramics. J Mater Process Technol 2008;206:221–30.

[72] Kinderlehrer D. Mathematical physics: added dimensions to grain growth. Nature 2007;446:995–6. 17460656 10.1038/446995a

[73] Descamps M , Boilet L, Moreau G, Tricoteaux A, Lu J, Leriche A, Lardot V, Cambier F. Processing and properties of biphasic calcium phosphates bioceramics obtained by pressureless sintering and hot isostatic pressing. J Eur Ceram Soc 2013;33:1263–70.

[74] Wang X , Fan H, Xiao Y, Zhang X. Fabrication and characterization of porous hydroxyapatite/β-tricalcium phosphate ceramics by microwave sintering. Mater Lett 2006;60:455–8.

[75] Parhi P , Ramanan A, Ray AR. A convenient route for the synthesis of hydroxyapatite through a novel microwave-mediated metathesis reaction. Mater Lett 2004;58:3610–2.

[76] Li L , Wang F, Liao Q, Wang Y, Zhu H, Zhu Y. Synthesis of phosphate based glass-ceramic waste forms by a melt-quenching process: the formation process. J Nucl Mater 2020;528:3–10.

[77] Lóh NJ , Simão L, Faller CA, Noni AD, Montedo ORK. A review of two-step sintering for ceramics. Ceram Int 2016;42:12556–72.

[78] Chu MY , Jonghe LC, Lin MK, Lin FJ. Precoarsening to improve microstructure and sintering of powder compacts. J Am Ceram Soc 1991;11:2902–11.

[79] Wang XH , Chen IW. Sintering dense nanocrystalline ceramics without final-stage grain growth. Nature 2000;404:168–71. 10724165 10.1038/35004548

[80] Jarcho M , Bolen CH, Thomas MB, Bobick J, Kay JF, Doremus RH. Hydroxylapatite synthesis and characterization in dense polycrystalline form. J Mater Sci 1976;11:2027–35.

[81] Thangamani N , Chinnakali K, Gnanam FD. The effect of powder processing on densification, microstructure and mechanical properties of hydroxyapatite. Ceram Int 2002;28:355–62.

[82] Montufar EB , Casas-Luna M, Tkachenko S, Fohlerova Z, Diaz S, Dvorak K, Celko L, Kaiser J. High strength, biodegradable and cytocompatible alpha tricalcium phosphate-iron composites for temporal reduction of bone fractures. Acta Biomater 2018;70:293–303. 29432984 10.1016/j.actbio.2018.02.002

[83] Ortali C , Julien I, Vandenhende M, Drouet C, Champion E. Consolidation of bone-like apatite bioceramics by spark plasma sintering of amorphous carbonated calcium phosphate at very low temperature. J Eur Ceram Soc 2018;38:2098–109.

[84] Shao J , Li W, Kou H, Deng Y. Temperature dependent fracture toughness model for whisker-reinforced ceramic matrix composites. J Am Ceram Soc 2022;105:4348–59.

[85] Gao C , Liu T, Shuai C, Peng S. Enhancement mechanisms of graphene in nano-58S bioactive glass scaffold: mechanical and biological performance. Sci Rep 2014;4:4712. 24736662 10.1038/srep04712 PMC3988481

[86] Jin HB , Oktar FN, Dorozhkin S, Agathopoulos S. Sintering behavior and properties of reinforced hydroxyapatite/TCP biphasic bioceramics with ZnO-whiskers. J Compos Mater 2011;45:1435–45.

[87] Feng C , Wu Y, Cao Q, Li X, Zhu X, Zhang X. Effect of hydrothermal media on the in-situ whisker growth on biphasic calcium phosphate ceramics. Int J Nanomed 2021;16:147–59. 10.2147/IJN.S280130 PMC7804068 33456309

[88] Robles-Águila MJ , Reyes-Avendaño JA, Mendoza ME. Structural analysis of metal-doped (Mn, Fe, Co, Ni, Cu, Zn) calcium hydroxyapatite synthetized by a sol-gel microwave-assisted method. Ceram Int 2017;43:12705–9.

[89] Safarzadeh M , Ramesh S, Tan CY, Chandran H, Noor AFM, Krishnasamy S, Johnson U, Ramesh S. Effect of multi-ions doping on the properties of carbonated hydroxyapatite bioceramic. Ceram Int 2019;45:3473–7.

[90] Basu S , Basu B. Doped biphasic calcium phosphate: synthesis and structure. J Asian Ceram Soc 2019;7:265–83.

[91] Albayrak O. Structural and mechanical characterization of boron doped biphasic calcium phosphate produced by wet chemical method and subsequent thermal treatment. Mater Charact 2016;113:82–9.

[92] Sopyan I , Gunawan Shah QH, Mel M. Fabrication and sintering behavior of zinc-doped biphasic calcium phosphate bioceramics. Mater Manuf Process 2016;31:713–8.

[93] Sprio S , Dapporto M, Preti L, Mazzoni E, Iaquinta MR, Martini F, Tognon M, Pugno NM, Restivo E, Visai L, Tampieri A. Enhancement of the biological and mechanical performances of sintered hydroxyapatite by multiple ions doping. Front Mater 2020;7:1–18.

[94] Liu Y , Huang J, Li H. Synthesis of hydroxyapatite-reduced graphite oxide nanocomposites for biomedical applications: oriented nucleation and epitaxial growth of hydroxyapatite. J Mater Chem B 2013;1:1826–34. 32261148 10.1039/c3tb00531c

[95] Siddiqui HA , Pickering KL, Mucalo MR. A review on the use of hydroxyapatite- carbonaceous structure composites in bone replacement materials for strengthening purposes. Materials (Basel) 2018;11:1813. 30249999 10.3390/ma11101813 PMC6212993

[96] Herkendell K , Shukla VR, Patel AK, Balani K. Domination of volumetric toughening by silver nanoparticles over interfacial strengthening of carbon nanotubes in bactericidal hydroxyapatite biocomposite. Mater Sci Eng C 2014;34:455–67. 10.1016/j.msec.2013.09.034 24268282

[97] Baradaran S , Moghaddam E, Nasiri-Tabrizi B, Basirun WJ, Mehrali M, Sookhakian M. Characterization of nickel-doped biphasic calcium phosphate/graphene nanoplatelet composites for biomedical application. Mater Sci Eng C 2015;49:656–68. 10.1016/j.msec.2015.01.050 25686995

[98] Wang X , Xue J, Ma B, Wu JF, Chang J, Gelinsky M, Wu C. Black bioceramics: combining regeneration with therapy. Adv Mater 2020;32:1–9. 10.1002/adma.202005140 33094493

[99] Sopyan I , Nawawi N, Shah Q, Ramesh S, Tan CY., Hamdi M. Sintering and properties of dense manganese-doped calcium phosphate bioceramics prepared using sol-gel derived nanopowders. Mater Manuf Process 2011;26:908–14.

[100] Ramesh S , Tan CY, Peralta CL, Teng WD. The effect of manganese oxide on the sinterability of hydroxyapatite. Sci Technol Adv Mater 2007;8:257–63.

[101] Tan CY , Ramesh S, Tolouei R, Sopyan I, Teng WD. Synthesis of high fracture toughness of hydroxyapatite bioceramics. Adv Mater Res 2011;264–265:1849–55.

[102] Sarkar SK , Youn MH, Oh IH, Lee BT. Fabrication of CNT-reinforced HAp composites by spark plasma sintering. Mater Sci Forum 2007;534–536:893–6.

[103] Guo L , Xin H, Luo X, Zhang C. Phase evolution, mechanical properties and MRI contrast behavior of GdPO_4_ doped hydroxyapatite for dental applications. Mater Sci Eng C 2020;111:110858. 10.1016/j.msec.2020.110858 32279759

[104] Fakhraei O , Hesaraki S, Alizadeh M. Fracture toughness and R-curve behavior of BCP/YTZP nanocomposites produced using the spark plasma sintering process. J Alloys Compounds 2017;725:623–31.

[105] Dwivedi VK , Dwivedi SP, Yadav R. Effect of heat treatment process on microstructure and mechanical behaviour of Al/MgO composite material. Adv Mater Process Technol 2022;8:653–62.

[106] Takagi Y , Nagata H, Takenaka T. Effects of quenching on bending strength and piezoelectric properties of (Bi_0.5_Na_0.5_)TiO_3_ ceramics. J Asian Ceram Soc 2020;8:277–83.

[107] Deshpande VK , Shankar J. Effect of heat treatment schedule on the properties of lead titanate based glass ceramic. Ferroelectrics 2009;393:63–70.

[108] Morozova LV , Kalinina MV, Panova TI, Popov VP, Drozdova IA, Shilova OA. Synthesis of the study of solid solutions based on the ZrO_2_-HfO_2_-Y_2_O_3_(CeO_2_) system. Glas Phys Chem 2017;43:464–70.

[109] Chen J , Tan L, Yang K. Effect of heat treatment on mechanical and biodegradable properties of an extruded ZK60 alloy. Bioact Mater 2017;2:19–26. 29744407 10.1016/j.bioactmat.2016.12.002 PMC5935012

[110] Bao M , Liu Y, Wang X, Yang L, Li S, Ren J, Qin G, Zhang E. Optimization of mechanical properties, biocorrosion properties and antibacterial properties of wrought Ti-3Cu alloy by heat treatment. Bioact Mater 2018;3:28–38. 29744440 10.1016/j.bioactmat.2018.01.004 PMC5935767

[111] Wątroba M , Bednarczyk W, Kawałko J, Bała P. Fine-tuning of mechanical properties in a Zn-Ag-Mg alloy via cold plastic deformation process and post-deformation annealing. Bioact Mater 2021;6:3424–36. 33817418 10.1016/j.bioactmat.2021.03.017 PMC7988494

[112] Liu Y , Huang J, Niinomi M, Li H. Inhibited grain growth in hydroxyapatite-graphene nanocomposites during high temperature treatment and their enhanced mechanical properties. Ceram Int 2016;42:11248–55.

[113] Shao Y , Song F, Liu B, Li W, Li L, Jiang C. Observation of ceramic cracking during quenching. J Am Ceram Soc 2017;100:520–3.

[114] Collin M , Rowcliffe D. Analysis and prediction of thermal shock in brittle materials. Acta Mater 2000;48:1655–65.

[115] Meyers MA , Chen PY, Lin A, Seki Y. Biological materials: structure and mechanical properties. Prog Mater Sci 2008;53:1–206. 10.1016/j.jmbbm.2008.02.003 19627786

[116] Gao HL , Chen SM, Mao LB, Song ZQ, Yao HB, Colfen H, Luo XS, Zhang F, Pan Z, Meng YF, Ni Y, Yu SH. Mass production of bulk artificial nacre with excellent mechanical properties. Nat Commun 2017;8:287. 28821851 10.1038/s41467-017-00392-z PMC5562756

[117] Sadowska JM , Guillem-Marti J, Montufar EB, Espanol M, Ginebra MP. Biomimetic versus sintered calcium phosphates: the in vitro behavior of osteoblasts and mesenchymal stem cells. Tissue Eng Part A 2017;23:1297–309. 28107811 10.1089/ten.TEA.2016.0406

[118] Zhou C , Ye X, Fan Y, Ma L, Tan Y, Qing F, Zhang X. Biomimetic fabrication of a three-level hierarchical calcium phosphate/collagen/hydroxyapatite scaffold for bone tissue engineering. Biofabrication 2014;6:035013. 24873777 10.1088/1758-5082/6/3/035013

[119] Liang Q , Deng C. Mechanical properties and preparation strategies of bone repair materials in the treatment of large bone defects. Mater Reports 2021;35:13100–8.

[120] Moosavifar M , Parsaei H, Hosseini SJ, Mirmontazeri SM, Ahadi R, Ahadian S, Engel FB, Roshanbinfar K. Biomimetic organic-inorganic nanocomposite scaffolds to regenerate cranial bone defects in a rat animal model. ACS Biomater Sci Eng 2022;8:1258–70. 35193354 10.1021/acsbiomaterials.1c01331

[121] Miao X , Tan LP, Tan LS, Huang X. Porous calcium phosphate ceramics modified with PLGA-bioactive glass. Mater Sci Eng C 2007;27:274–9.

[122] Xu M , Zhai D, Xia L, Li H, Chen S, Fang B, Chang J, Wu C. Hierarchical bioceramic scaffolds with 3D-plotted macropores and mussel-inspired surface nanolayers for stimulating osteogenesis. Nanoscale 2016;8:13790–803. 27380634 10.1039/c6nr01952h

[123] Wu C , Han P, Liu X, Xu M, Tian T, Chang J, Xiao Y. Mussel-inspired bioceramics with self-assembled Ca-P/polydopamine composite nanolayer: preparation, formation mechanism, improved cellular bioactivity and osteogenic differentiation of bone marrow stromal cells. Acta Biomater 2014;10:428–38. 24157695 10.1016/j.actbio.2013.10.013

[124] Tang Y , Wang J, Cao Q, Chen F, Wang M, Wu Y, Chen X, Zhu X, Zhang X. Dopamine/DOPAC-assisted immobilization of bone morphogenetic protein-2 loaded heparin/PEI nanogels onto three-dimentional printed calcium phosphate ceramics for enhanced osteoinductivity and osteogenicity. Biomater Adv 2022;140:213030. 36027668 10.1016/j.bioadv.2022.213030

